# A prospective interventional study of recombinant human interleukin-11 mouthwash in chemotherapy-induced oral mucositis

**DOI:** 10.1186/s12903-022-02348-2

**Published:** 2022-07-29

**Authors:** Hangping Wei, Juan Wei, Xiaofang Dong

**Affiliations:** Department of Medical Oncology, Affiliated Dongyang Hospital of Wenzhou Medical University, No. 60 West Wuning Road, Dongyang, 322100 Zhejiang People’s Republic of China

**Keywords:** Oral mucositis, Recombinant human interleukin-11, Chemotherapy, Treatment, Prevention

## Abstract

**Background:**

This prospective interventional study aimed to evaluate and analyse the efficacy of rhIL-11 mouthwash compared to Kangfuxin fluid in treatment and blank control in prevention of oral mucositis (OM) in patients receiving chemotherapy.

**Materials and Methods:**

In total, 50 patients in the treatment group and 62 patients in the prevention group were included. Subsequently, each group was divided into an experimental group and a control group. In the treatment group, the experimental patients received recombinant human interleukin-11 (rhIL-11) mouthwash, whereas the control group received Kangfuxin fluid. In the prevention group, experimental patients still received rhIL-11 mouthwash based on routine oral care, whereas the control group only received routine oral care. Meanwhile, we observed and recorded the efficacy in the treatment group, and the occurrence and grades of OM in the prevention group.

**Results:**

Through statistical analysis, the results showed that on the seventh day of treatment, the experimental group showed more improvement compared to the control group, and it was statistically significant (*p* = 0.032). The average healing time in the experimental group (3.59 ± 1.927 days) was shorter than that in the control group (4.96 ± 2.421 days; *p* = 0.031). In the prevention group, we observed the incidence of oral mucositis. No significant differences were found in the occurrence and grades of OM in the experimental and control groups (*p* = 0.175).

**Conclusion:**

Our preliminary results indicate that rhIL-11 mouthwash may be a superior option to treat OM, especially in severe cases, compared to Kangfuxin fluid. However, there is no advantage in prevention.

## Background

Oral mucositis (OM) is a frequent complication of cancer treatment toxicities, occurring in approximately 20–60% of cancer patients receiving conventional antineoplastic therapies [1–3]. Previous research demonstrates that the pathophysiology of OM is a complex, multistage phenomenon that can result from oral mucosal cell damage caused by free radicals produced by chemotherapy drugs [4]. It involves amplified inflammatory responses, reduced cell proliferation, increased cell senescence/apoptosis, and impaired regenerative potential in both the mucosal and submucosal compartments [1, 5, 6].Lesions may be erosive or ulcerative and can cause mild to severe pain. Although the condition is self-limiting, it usually leads to a significant decrease in the quality of life due to prolonged hospital stay, altered nutritional status, increased risk of infection, and increased prescription of opioids. Moreover, it has a negative impact on the delivery of optimal cancer treatments [5, 6]. For these reasons, it is extremely necessary to treat OM, with the goal of preventing or reducing the severity of lesions and managing the associated symptoms, thereby allowing the continuation of cancer therapy [1, 7, 8].

Recently, a growing number of OM remedies has been suggested in the literature, including basic oral care, antimicrobial agents, anaesthetics and analgesics, laser therapy, and oral cryotherapy [1, 8]. Basic oral care is considered the backbone of supportive care for patients receiving cancer treatment, which could reduce the incidence of moderate to severe OM [4, 8]. At present, mixed medication mouthwashes, which include antibacterial drugs, analgesics, vitamin B12, Xilei powder and hormone therapy, are widely used clinically [9, 10].

The glycoprotein recombinant human interleukin-11 (rhIL-11) is a pleiotropic cytokine that exhibits diverse therapeutic effects [11]. It has been widely used for treating bone marrow suppression caused by chemotherapy and radiotherapy since its discovery in a mouse lung-conditioned medium in 1977 [12]. Recent studies have found that rhIL-11 can directly promote the proliferation and migration of endothelial cells, enhance the activity of other angiogenic factors, and promote healing of the mucosa [7, 13]. Currently, there are a few articles focusing on the value of rhIL-11 in OM, and there is no strong evidence that rhIL-11 can be recommended for the prevention and treatment of OM.

Therefore, we predict that rhIL-11, combined with normal saline, can directly act on oral epithelial cells when used as an oral wash and promote the healing of OM by accelerating the proliferation of oral epithelial cells. The purpose of this research is to prospectively evaluate whether rhIL-11 is more effective, compared to Kangfuxin fluid in treatment and blank control in prevention of oral mucositis (OM) in patients receiving chemotherapy.

## Materials and methods

### Patients

This prospective interventional study was a randomized control trial conducted from January 2019 to March 2022 on patients receiving chemotherapy in the Department of Oncology at Affiliated Dongyang Hospital of Wenzhou Medical University. Patients older than 18 years; those receiving non-systemic medication; those with previous head and neck radiotherapy time of less than 1 year; those with allergies, oral lesions, or serious illnesses (transaminase more than twice the normal value, renal failure, and infection, among others); or an Eastern Cooperative Oncology Group performance status score of > 2 were excluded. Those who failed to gargle as directed were also excluded. The study rationale was explained to the trial participants, and written informed consent was obtained prior to enrolment. Finally, 50 patients in the treatment group and 62 patients in the prevention group completed the study and were analysed.

### Study design

Patients who would receive a systemic chemotherapy were assigned to the prevention group (n = 62) and were selected using a random distribution into the experimental group (n = 34) and control group (n = 28). A random number table was used for random distribution. Patients in the experimental group received rhIL-11 mouthwash (3 mg lyophilised powder of rhIL-11, which was dissolved in distilled water and added to 100 mL normal saline) based on routine oral care (brushing teeth, gargling, and daily oral hygiene monitoring) during chemotherapy, whereas the control group only received routine oral care. Patients were instructed to gargle 10 mL of solution four times per day (morning, lunch, after dinner, and before going to bed) for 2–3 min each time.

Patients with OM of degree II or above after the current round of chemotherapy were assigned to the treatment group (n = 50) and were selected using a random distribution into the experimental (n = 27) and control groups (n = 23). A random number table was also used for random distribution. Patients in the experimental group received rhIL-11 mouthwash (3 mg lyophilised powder of rhIL-11, which was dissolved in distilled water and added to 100 mL normal saline) compared to Kangfuxin fluid (its main chemical component is *Periplaneta americana* extract, a liquid drug that can significantly promote angiogenesis and growth of granulation tissue, accelerate the shedding of necrotic tissue, and rapidly repair ulcers and wounds; it has been used widely used in clinic and has been shown the effect in the treatment of oral mucositis [14–17]) in the control group in those patients with OM after chemotherapy. Instructions for the two mouthwashes were the same as those for the prevention group. The ulcer was also smeared with a cotton ball, and treatment continued until the ulcer healed. Local treatment helps to accelerate the healing of oral mucositis.

### Evaluation

The scale proposed by the World Health Organization classifies OM in five degrees: Grade 0, incipient and asymptomatic lesions; Grade I, slight oral soreness and erythema; Grade II, oral erythema, small ulcers, solid diet tolerated; Grade III, large oral ulcers, liquid diet only; Grade IV, severe ulcers, bleeding, pain, and oral alimentation impossible. On this scale, Grades III and IV correspond to severe OM. Oral mucositis healing refers to OM of degree 0, that is, the elimination of ulcers and reduction of pain.

Every morning before administration, medication record, curative effect observation, and effect evaluation were made by specialised staff (a trained nurse blinded to the intervention), and the injury site, ulcer area size, ulcer number, and pain degree were evaluated according to the OM grading standards. The treatment group was followed up to the basic healing of oral mucositis, and the preventive group was followed up to the beginning of the next cycle of chemotherapy.

### Ethics approval and consent to participate

The study has been approved by the ethics committee of our hospital.

### Statistical analysis

The clinical data were described using medians, frequencies, and percentages. SPSS 22.0 software was used for statistical analysis. The measurement data were analysed using the T-test, and counting data were analysed using the chi-square test or wilcoxon test. A *p* value of < 0.05 was considered statistically significant.

## Results

### Efficacy of rhIL-11 in the treatment group

#### Patient demographics and baseline characteristics

In the experimental and control groups, the ratio of males to females was 1.17:1, and the median age was 63.6 years (range, 26–81 years). The patients included were mostly undergoing treatment for thoracic and abdominal tumours, such as lung and intestinal cancer. The majority comprised stage IV patients who had received chemotherapy previously. There was no significant difference in general characteristics among the groups (*p* > 0.05) (Table 1).

**Table 1:** Patient characteristics in the treatment group

Characteristics	Experimental group	Control group	*p* value
	n (%)	n (%)	
Age			*p* = 0.643
≥ 65 years	17(63)	13(56.5)	
< 65 years	10(37)	10(43.5)	
Gender			*p* = 0.811
Male	15(55.6)	12(52.2)	
Female	12(44.4)	11(47.8)	
Tumour site			*p* = 0.817
Head and neck	3(11.1)	2(8.7)	
Chest	10(37.0)	10(43.5)	
Abdomen	11(40.7)	10(43.5)	
Other	3(11.1)	1(4.3)	
Disease stage			*p* = 0.167
II	1(3.7)	0(0)	
III	7(25.9)	2(8.7)	
IV	19(70.4)	21(91.3)	
PS			*p* = 0.403
0	0(0)	1(4.3)	
1	20(74.1)	14(60.9)	
2	7(25.9)	8(34.8)	
Chemotherapeutic drugs			*p* = 0.475
Platinum ± 5-FU	9(33.3)	5(21.7)	
Paclitaxel	8(29.6)	11(47.8)	
EN Huan	2(7.4)	2(8.7)	
Target	6(22.2)	2(8.7)	
Other	2(7.4)	3(13)	
Previous chemotherapy			*p* = 0.114
Yes	20(74.1)	21(91.3)	
No	7(25.9)	2(8.7)	
Previous radiotherapy			*p* = 0.857
Yes	4(14.8)	3(13)	
No	23(85.2)	20(87)	

#### Comparison of the efficacy of experimental and control groups

OM in this study was predominantly classified as stage II, accounting for 76% of all cases, and the severity of OM before treatment in the two groups was not statistically significant (*p* = 0.696) (Table 2). On the second and fourth days of treatment, there was no significant improvement in the two groups. (*p*  = 0.793, 0.069) (Table 2). However, on the seventh day of treatment, the experimental group showed more improvement compared to the control group, and it was statistically significant (*p* = 0.032) (Table 2). The average healing time was 3.59 ± 1.927 days in the experimental group and 4.96 ± 2.421 days in the control group (*p* = 0.031) (Table 3). As a result, among all the treatment patients, the efficacy was higher in the experimental group than in the control group. The average healing time of the experimental group was shorter than that of the control group.

**Table 2:** Oral mucositis in the experimental and control groups on the second, fourth, and seventh day of treatment within the treatment group (the wilcoxon test was adopted)

Classification of oral mucositis	Before treatment		D2		D4		D7	
	Experimental group (n)	Control group (n)	Experimental group (n)	Control group (n)	Experimental group (n)	Control group (n)	Experimental group (n)	Control group (n)
0	0	0	2	0	15	7	24	15
I	0	0	1	4	8	10	3	4
II	21	17	19	13	3	2	0	1
III	4	3	5	5	1	4	0	3
IV	2	3	0	1	0	0	0	0
P value	*p* = 0.696		*p* = 0.793		*p* = 0.069		*p* = 0.032

**Table 3:** Healing rate of the experimental and control groups within the treatment group

Treatment	No. of patients	Healing time (days)	T value	*p* value
Experimental group	27	3.59 ± 1.927	2.218	0.031
Control group	23	4.96 ± 2.421		

### Efficacy of rhIL-11 in the prevention group

#### Patient demographics and baseline characteristics

The ratio of males to females was 1.21:1, and the median age was 62.6 years. Most of them (98.3%) were thoracic or abdominal tumors and were in the advanced stage of the disease. There was no difference in gender, age, disease location, chemotherapeutic drugs and disease condition between the two groups (Table 4, *p* > 0.05).

**Table 4:** Patient characteristics in the prevention group

Characteristics	Experimental group	Control group	*p* value
	n (%)	n (%)	
Age			*p* = 0.368
≥ 65 years	16(47.1)	10(35.7)	
< 65 years	18(52.9)	18(64.3)	
Gender			*p* = 0.368
Male	16(47.1)	10(35.7)	
Female	18(52.9)	18(64.3)	
Tumour site			*p* = 0.311
Head and neck	0(0)	1(3.6)	
Chest	15(44.1)	17(60.7)	
Abdomen	18(52.9)	9(32.1)	
Other	1(2.9)	1(3.6)	
PS			*p* = 0.990
0	4(11.8)	3(10.7)	
1	7(20.6)	6(21.4)	
2	23(67.6)	19(67.9)	
Chemotherapeutic drugs			*p* = 0.857
Platinum ± 5-FU	15(44.1)	16(57.1)	
Paclitaxel	10(29.4)	7(25)	
EN Huan	1(2.9)	1(3.6)	
Target	6(17.6)	3(10.7)	
Other	2(5.9)	1(3.6)	

#### Comparison of the experimental and control groups in the prevention group

The OM incidence rates were 11.8% and 25% in the experimental and control groups, respectively (Chi square test was used according to the incidence of OM, *p* = 0.175). In order to further analyse the severity of oral mucositis, the wilcoxon test was used (*p* = 0.16 Table 5). As a result, the occurrence and grades of OM in the experimental and control group showed no significant differences.

**Table 5:** OM incidence rate in the experimental and control groups within the prevention group

Prevention	No. of patients	Classification of oral mucositis					Incidence rate
		0	I	II	III	IV	
Experimental group	34	30(88.2%)	3(8.8%)	1(3%)	0(0%)	0(0%)	11.8%
Control group	28	21(75%)	4(14.3%)	2(7.1%)	1(3.6%)	0(0%)	25.0%
P value		*p* = 0.16 (the wilcoxon test was adopted)					*p* = 0.175

## Discussion

OM is a common adverse effect of anticancer therapy, typically occurring within 1 week of undergoing chemotherapy. In addition to causing discomfort and pain, OM may affect the quality of life of cancer patients by prolonging hospitalisation and increasing financial expenses. Therefore, it is increasingly important to understand the mechanisms of OM lesions to provide effective prevention and treatment. Clinically, different chemotherapy regimens can cause different degrees of OM. Current research indicates that antimetabolites, which affect DNA synthesis, are associated with 40–60% of OM incidences, with the use of 5-FU and platinum derivatives (cisplatin and oxaliplatin) resulting in more severe OM [18–20]. Although no evidence for a cumulative effect with repeated chemotherapy cycles has been found, considering the use of the same drugs and the sensitivity of the body, it remains necessary to consider using prophylactic drugs for patients with a history of severe OM [21].

Prevention and treatment of OM is necessary to relieve symptoms, accelerate tissue repair, and promote successful chemotherapy. Many treatments, including basic oral care, antimicrobial agents, and natural medicine using substances such as honey, aim to reduce both the incidence and severity of OM [2, 10, 22, 23]. However, these treatments have their own limitations, including cariogenic effects and adherence to teeth following prolonged ingestion of honey [24]. Previous studies suggest that preventive oral spray can delay the occurrence of OM [9, 14]. Some studies showed that laser therapy and cryotherapy reduced the incidence of severe oral mucositis and reduced pain [14]. Though much research has been done on the role of cytokines (mostly GM-CSF and G-CSF) in oral mucositis, results have been inconsistent [25–27].

RhIL-11 is a pleiotropic cytokine which stimulates myeloid, erythroid, and megakaryocyte differentiation, modulates macrophage and T cell inflammatory functions, and protects the function of the mucosal epithelium [11, 12, 21]. It is also known to promote mitosis and proliferation by inhibiting apoptosis and improving the mitotic activity of small intestinal villi stem progenitor cells, and it has a good protective effect on gastrointestinal tract injuries caused by radiotherapy and chemotherapy. The oral mucosa is similar to the intestinal mucosa in terms of continuous self-renewal and rapid proliferation [28]. Thus, rhIL-11 has the potential to reduce chemotherapy-related OM. Moreover, epithelial cells contain the colony-stimulating factor receptor, which is bound by rhIL-11 to stimulate the migration and proliferation of epithelial cells and promote the growth of keratinocytes and fibroblasts [7]. Thus, rhIL-11 may promote the proliferation and maturation of endothelial cells to accelerate the healing of OM caused by chemotherapy.

Our data were collected prospectively, eliminating potential recall bias and increasing credibility compared to a retrospective design. Prior research in an animal model found that rhIL-11 favourably modulates acute radiation-induced mucositis by attenuating pro-inflammatory cytokine expression [29]. Unfortunately, there is no relevant prospective research to support these findings. In the present study, the results showed that rhIL‐11 therapy significantly reduced the recovery time of OM (3.59 ± 1.927 days vs. 4.96 ± 2.421 days; *p* = 0.031) and improved the efficiency of treatments compared with Kangfuxin fluid, supporting the ability of rhIL‐11 to accelerate recovery from OM. In addition, this study demonstrates there was no significant difference in the efficacy of rhIL-11 or Kangfuxin fluid in the first few days, but after 1 week of treatment, the efficacy of rhIL-11 in OM was better than Kangfuxin fluid. This further indicates that rhIL-11 may shorten the treatment time of severe OM. This study also found that no advantage of rhIL-11 treatment in preventing OM, both the incidence and degree of oral mucositis. At present, many studies have shown that low-intensity laser therapy, honey and probiotics play a role in prevention.

However, there are few a samples after radiotherapy in this study. According to previous studies, the incidence of OM related to radiotherapy of head and neck tumours is high and the treatment time is long, which need to be further investigated. Moreover, the severity of chemotherapy-induced OM in this study is low, which may offset the results. In addition, the main limitation of our experiment is the small sample size of this study; therefore, further high‐quality, large‐scale studies are needed to verify our conclusions.

## Conclusions

In summary, our preliminary results indicate that recombinant human interleukin-11 mouthwash may be a superior option option to treat OM, especially in severe cases, compared to Kangfuxin fluid. Considering the cost of rhIL-11, recombinant human interleukin-11 mouthwash is not recommended for preventing OM in patients receiving chemotherapy.

## Data Availability

All data generated or analysed during this study are included in this published article. And the primary data could be achieved from the corresponding author.

## Abbreviations

OM: Oral mucositis
rhIL-11: Recombinant human interleukin-11
PS: Performance status
